# Sleep and Neuroimmunomodulation for Maintenance of Optimum Brain Function: Role of Noradrenaline

**DOI:** 10.3390/brainsci12121725

**Published:** 2022-12-16

**Authors:** Rachna Mehta, Rohosen Bhattacharya, Birendra Nath Mallick

**Affiliations:** Amity Institute of Neuropsychology & Neurosciences, Amity University, Noida 201301, India

**Keywords:** neuroinflammation, microglia, noradrenaline, sleep loss

## Abstract

Immune function and sleep are two normal physiological processes to protect the living organism from falling sick. There is hardly any disease in which they remain unaffected, though the quantum of effect may differ. Therefore, we propose the existence of a strong correlation between sleep (quality or quantity) and immune response. This may be supported by the fact that sleep loss modulates many of the immunological molecules, which includes interferons; however, not much is known about their mechanism of action. Sleep is divided into rapid eye movement sleep (REMS) and non-REMS. For practical reasons, experimental studies have been conducted mostly by inducing loss of REMS. It has been shown that withdrawal of noradrenaline (NA) is a necessity for generation of REMS. Moreover, NA level increases in the brain upon REMS loss and the elevated NA is responsible for many of the sleep loss-associated symptoms. In this review, we describe how sleep (and its disturbance/loss) modulates the immune system by modulating the NA level in the brain or vice versa to maintain immune functions, physiological homeostasis, and normal healthy living. The increased levels of NA during REMS loss may cause neuroinflammation possibly by glial activation (as NA is a key modulator of microglia). Therefore, maintaining sleep hygiene plays a crucial role for a normal healthy living.

## 1. Introduction

Living organisms are continuously challenged by environmental factors. In the process, the output or products of many physiological processes contribute to maintaining these physiological processes at equilibrium resulting in normal healthy living. However, if these processes get disturbed, the state of equilibrium is altered, and one may become sick or diseased. For survival, our physiological processes withstand or negotiate with the harmful molecules by restricting their entry into the body or by neutralizing them as such or the byproducts produced upon their reaction with other systems [1,2]. However, if they entered, the body may eliminate the molecule(s) through excretion or by releasing some byproduct(s). Nevertheless, some may multiply within the body and affect the physiological process(es). Many such toxic substances may challenge the immune system of the body, which in turn uses a wide array of mechanisms to control, normalize, and eliminate the causative factors. While doing this, the immune system must possess the ability to discriminate between the pathogen (or the unwanted molecule) and that of the host cells to avoid self-destruction [1]. Loss of such discrimination and failure of self-tolerance may result in autoimmune disease. The activation of immune system shifts the equilibrium of the physiological processes by enhancing one or more such factors, including interferons (IFN), cytokines, macrophages, and monocytes, which work together to restore the body’s normal condition [3]. Classically, these are the innate and adaptive immune responses raised by the immune system against pathogenic invasion in our body [4].

The immune system functions in close association with the nervous system. Several studies have shown production of immune factors by the brain and neuroendocrine mediators by the immune system [5,6]. These immune factors include chemokines, growth factors, enkephalins, endorphins, neurotrophic peptides, etc. There is evidence for the presence and expression of cytokines, Toll-like receptors, the molecules of complement family, the major histocompatibility complex, and receptors of antibodies in the lymphatic vessels and the brain. Interestingly, these have been shown to play crucial role in brain development. Lymphocytes along with microglia are known to play a pivotal role in the formation of neuronal circuits and regulate cognition [7]. Besedovsky et al. [8] inferred the communication between immune system and brain by demonstrating activation of the hypothalamic–pituitary–adrenal axis and the sympathetic nervous system during the peak antibody response in mice vaccinated with a T-cell antigen. The immune cells regulate the functioning of the central nervous system by regulating synaptic plasticity, both during development as well as at adulthood [9]. A long-range interaction between the two permits the nervous system to modulate the immune response(s) in its fight against infection from pathogenic microorganisms or foreign molecule. Immunogenic challenge(s) may damage living cells and cause release of prostaglandins, an inflammatory response [4], which may modulate thermoregulatory system [10] inducing changes in body temperature. In the process, locally, the brain cells may produce cytokines, which in turn may be responsible for the interactions between endothelial cells, glia, and neurons [11]. Microglia, the resident immune cells in the central nervous system, are known to monitor the brain for tissue damage and respond by modulating tissue homeostasis [12].

Thus, immune response is an inherent, instinct property of the brain to perform optimally for maintenance of an equilibrium state necessary for leading normal healthy living [1]. Disturbance or a shift in this equilibrium might result into a dysfunction, an altered state, or a disease. If protection is not possible, a recovery from such disturbances is crucial to avoid irreversible damage. Sleep is one such fundamental, instinctive, and natural remedial phenomenon which has evolved to maintain homeostasis to restore normal physiological processes and healthy living. Sleep is affected by many internal factors, environment as well as lifestyle changes. The modern lifestyle threatens the sleep behavior and its pattern, which affects the health negatively. Therefore, it has been proposed that a disciplined sleep habit is essential for healthy living [13]. A disciplined sleep habit includes sleep hygiene, sleep routine e.g., following time going to bed and waking time, sleeping environment e.g., bedroom lighting, bedding conditions, quality, and quantity of food, etc. Sleep has been broadly divided into rapid eye movement sleep (REMS) and non-REMS (NREMS). One spends the least time in REMS, which repeats a few times in a normal human being [14]. REMS is characterized by rapid movement of the eyes, increased brain activity, and loss of muscle tone; one often dreams during this stage. Some amount of NREMS is necessary for the appearance of REMS. Therefore, in the absence of NREMS, REMS does not appear. Hence, loss of NREMS is practically comparable to total sleep (NREMS + REMS) loss; thus, often one studies the effect and functions of REMS and its loss [15,16].

REMS is postulated to perform the housekeeping functions of the brain and thus, maintains brain excitability, the fundamental property of the brain [17]. It is highest in newborn babies and gradually reduces with aging which signifies its importance in brain development and maturation. The quantity of REMS reduces with age, however, it is never absent in life [14,18]. Noradrenaline (NA) is one of the key bio-molecules whose level is lowest during REMS [19] and increases upon sleep loss, particularly during REMS loss, and it induces several REMS loss associated acute and chronic effects [19,20]. The release of NA from locus coeruleus (LC) neurons is modulated by gamma-amino butyric acid (GABA) for the regulation of REMS.

It has been proposed that in evolution, neuronal circuitry in the brain and the REMS came into existence possibly to maintain brain NA at optimum level and to protect the antioxidant compromised organ, the brain, from constant oxidative onslaught [21,22]. NA is known to increase arousal and alertness and promote vigilance in higher vertebrates; however, at elevated levels, it is known to induce multiple abnormalities at behavior and cellular levels including restlessness, anxiety, abnormal neuronal morphology, oxidative stress, and apoptosis [23,24,25]. The selective deprivation of REMS causes a significantly increased level of NA, which has been consistently studied and reported [19,24,26]. It has been shown that at a relatively lower level, NA exerts neuroprotection, while it is destructive at a higher concentration [25]. Combining this knowledge, it has been proposed that in evolution, REMS evolved to maintain the optimum level of NA in the brain as a normal physiological protective phenomenon [20].

As a signaling molecule, NA originated as octopamine in groups of neurons in ganglia of invertebrates, e.g., in Echinodermata, Mollusca, and Crustacea [27,28] and it is found to perform comparable functions as it does in vertebrates, although effects of its elevated levels in invertebrates are yet to be studied. Additionally, resembling REMS in vertebrates [29,30], REMS-like state has also been reported in several invertebrates [31,32]. Thus, the diversity and complexity of REMS has functional and evolutionary significance relating to species-specific patterns and emergence across development including the regulation of neurotransmitter (particularly NA) levels to maintain health and survival. In addition to NA-ergic neurons, cholinergic, GABA-ergic neurons are also involved in REMS regulation, while dopaminergic, orexinergic, and other neurons play a modulatory role [15]. The ventral portion of the sublaterodorsal nucleus of pons contains spinally projecting neurons whose activation induces motor atonia associated with REMS. Activation of the orexin-ergic neurons in the perifornical area has been reported to facilitate waking and reduce REMS. These neurons send dense projections to LC and activate the LC neurons to increase and decrease waking and REMS, respectively [33].

Sleep (including REMS) is considered an important modulator of immune response and its loss increases the susceptibility of an organism to infectious diseases [34,35]. NA is also reported to modulate the immune system [36,37] and plays a significant role in controlling the vulnerability to different types of infections [36]. Inflammatory responses can also be expressed due to disruptions of homeostatic processes as well as that of sleep [8]. Inflammation is often amongst the first physiological response of the body exposed to a challenge; the response may be acute or chronic. The former includes thermoregulatory, circulatory, cardio-vascular changes, while the latter includes sustained humoral changes. Both the acute as well as the chronic changes may be modulated by NA, which in turn is physiologically maintained by REMS. In recent days, COVID has affected humans across the globe and we are yet to understand its mechanism of action. Notwithstanding, it has been observed that COVID induces neuroinflammation [38] and many acute phase responses, e.g., increase in C-reactive protein (CRP), an acute phase response protein [39], IL6 [40], ferritin [41], ceruloplasmin [42], high fever [43], etc. In addition, isolated studies have reported that most of these factors might be modulated by NA [44,45,46,47,48,49,50,51]. Many patients have reported suffering from post-COVID symptoms much later after the classical COVID symptoms have subsided, e.g., cognitive and memory deficiencies [52], disturbed sleep [53], etc. We propose that it may be worthwhile to systematically correlate the sleep patterns and NA level in the COVID patients and their symptomatic treatment might bring much desirable results, at least in some cases.

In this review, we elaborate how sleep loss/disturbances, particularly REMS and its loss modulates immune system. Further, as REMS and its loss significantly modulates NA and it is responsible for many of the associated disorders and symptoms, we will emphasize how REMS loss-associated elevated NA modulates the immune responses.

## 2. Relationship between Sleep and Immune Function

Sleep is an essential physiological phenomenon present in species higher in the evolution. Although we are yet to completely unravel why we sleep, it is known that sleep conserves energy and adjusts the physiological processes to the challenges of the environment to maintain homeostasis in a living system [54]. Directly or indirectly, sleep affects or gets affected by most of the physiological processes controlled by the brain. In addition to this, sleep modulates several other physiological processes such as neuroplasticity, memory formation, energy maintenance, neuroprotection, etc. Studies over the past two decades have made a strong argument in favor of a bidirectional relationship between sleep and the immune system [35].

Sleep deprivation studies have reported a compromised immune functions and under- or over-secretion of cytokines [55]. Additionally, immune system regulators modulate different stages of sleep. The amount of time spent in NREMS increases, while REMS is reduced in cases of infections [56]. Shift workers and students studying overnight compromising sleep time have been seen to have increased propensity to suffer from common cold or flu, which suggests that sleep loss possibly enhances susceptibility to infections [13]. REMS loss affects several interleukins (ILs), neuronal structural proteins, and apoptosis in the brain [23,57], and initiates acute phase response proteins [58]. During normal sleep, circulating levels of IL-6 become more pronounced with an increase during REMS and decrease during NREMS [59]. Sleep deprivation experiments have shown that early-night sleep loss leaves IL-6 levels relatively unchanged, while late-night sleep deprivation is associated with under-secretion of IL-6; total sleep deprivation also diminishes the night-time IL-6 release by about half [60]. These suggest that IL-6 levels remain low during the early night, a period dominated by NREMS, while its levels increase during late night mostly during the REMS dominated period. Over-secretion of IL-6 levels has been observed in extremely long duration of sleep [61]. Daytime sleepiness and fatigue have also been associated with higher levels of IL-6. Therefore, it may be inferred that optimum sleep is necessary to maintain the IL-6 levels in plasma.

Tumor necrosis factor-α (TNF-α), which is released by microglia in the brain, has been shown to modulate sleep [62]. Administration of TNF-α inhibitors leads to an upsurge in REMS duration and reduces NREMS time [62]. The amount of sleep was seen to be closely related to number of white blood cells across 26 mammalian species. It was seen that those with more sleep had more white blood cell count favoring better immuno-competency [35]. The decreased number and activity of phagocytes, natural killer cells, and the white blood cells in the REMS-deprived animals suggest severely compromised or a weakened immune system [63]. Another immunogenic factor, IL-1β, possesses the capacity to enhance NREMS and reduces REMS. IL-1β acts directly on at least NA-ergic and serotonin-ergic neurons to regulate sleep [56]. It inhibits wake-promoting neurons in the preoptic area of the brain, an area known to regulate NREMS [64]. Inhibition of IL-1β by intracerebro-ventricular injection of IL-1β antagonist induces waking and reduces NREMS substantially. The level of CRP is known to increase during sleep disturbances [61]. However, although CRP is not a known mediator of sleep, it is a major indicator of sleep disturbances.

IFNs primarily act as endogenous antiviral agents in our body and are secreted by activation of interferon regulating factors. Both IFN-regulating factors and NF-κB are stimulated by macrophages [65]. IFNs are also potent somnogenic substances like IL-1 and TNF-α. Levels of IFNs increase upon sleep deprivation, like other cytokines as discussed above. IFNs promote sleep by stimulating IL-1 production [66], intracerebral and intravenous injection of IFN-α and IFN-β increase slow wave sleep without a reduction in REMS duration [67]. Thus, as discussed above, levels of various cytokines (IL-1β, TNF-α, etc.) get modified during sleep (which includes REMS) or its loss and their over-expression is usually considered as a sign of sleep disturbances. Therefore, there is enough convincing evidence to support that sleep and immune response have interdependencies. However, the mechanisms by which they influence each other are not completely understood.

## 3. Modulatory Role of Neurotransmitters on Immune Response

Neurotransmitters play a crucial role in maintaining sleep–wake cycles as well as immunity. It has been found that acetylcholine can improve cellular immunity and elevates early processes necessary for T-cell proliferation. In the central nervous system, acetylcholine has an immunoinhibitory function, while NA acts as an immunostimulatory [68]. GABA and acetylcholine have been shown to be effective anti-inflammatory regulators. For instance, it has been shown that GABA blocks NF-κB and p38 MAPK signaling pathways, thereby reducing the release of inflammatory cytokines [69]. GABA suppresses the reactive response of both astrocytes and microglia to the inflammatory stimulants, lipopolysaccharide, and interferon-γ, by inhibiting induction of inflammatory pathways mediated by NFκB and P38 MAP kinase. This causes decreased release of inflammatory cytokines TNFα and IL-6 and an attenuation of conditioned medium neurotoxicity. Acetylcholine inhibits cytokine production in the peripheral nervous system through the “cholinergic anti-inflammatory reflex” by binding to muscarinic receptors in the brain [70,71]. A potential reduction in GABA and acetylcholine release in the brain might encourage inflammatory responses [72]. Thus, GABA-ergic and cholinergic neurons which are active during REMS [15,19] contribute to maintenance of anti-inflammatory response during sleep.

The effects of cytokines and their signaling pathways on neurotransmitter systems such as serotonin, NA, dopamine, and glutamate have gained attention. The acute and sub-chronic effects of cytokines on the brain’s neurotransmitter systems are well documented [73]. Cytokines can affect neurotransmitter metabolism and potentially impair neurotransmitter function through a variety of ways, particularly, affecting their synthesis. For instance, IFN-α initiates a reaction that turns tryptophan into kynurenine, potentially reducing the amount of serotonin available in the brain [74]. Tetrahydrobiopterin disruption is another way by which inflammatory cytokines might affect the production of monoamine neurotransmitters. Tryptophan hydroxylase and tyrosine hydroxylase, the rate-limiting enzymes for the synthesis of serotonin, dopamine, and NA, respectively, require tetrahydrobiopterin as an important enzyme co-factor [72].

Inflammatory cytokines interact with serotonin and NA [56,75], and such interactions hint at the possible link between inflammation and sleep. Cytokines known to affect sleep include IL-1α, IL-1β, IL-2, IL-4, IL-6, IL-8, IL-10, IL-13, IL-15, IL-18, TNFα, TNFβ, IFNα, IFN-β, INF-γ, and macrophage inhibitory protein 1β [76,77]. IL-1 has been reported to release serotonin and NA in the hypothalamus [78]. This serotonin then inhibits cholinergic neurons and stimulates synthesis of IL-1, which inhibits wake-promoting neurons by enhancing the inhibitory effects of GABA and activating sleep promoting neurons in preoptic area [64,78,79]. IL-lβ may directly affect the neurons in anterior hypothalamus, which is supported by the presence of IL-lβ receptors on the neurons in the rat hypothalamus [80]. The possibility of IL-lβ activating neurons in other regions resulting into monoamine release cannot be ruled out. IL-lβ elicits synthesis and release of corticotropin releasing hormone in the median eminence near hypothalamus [81]. Direct infusion of corticotropin-releasing hormone into LC increases central and peripheral levels of catecholamines and their metabolites [82], possibly IL-l β enhances levels of NA in anterior hypothalamus by mediating the activation of LC neurons.

Sleep loss may trigger or worsen the prognosis of many diseases. Unsurprisingly, sleep disorders such as insomnia, or associated diseases e.g., narcolepsy, sleep-disordered breathing, etc., exacerbate existing ailments by compromising the immune system [83,84]. Improper or sub-optimal functioning of the immune system leaves the body vulnerable to many diseases. For instance, the role of cytokines has been strongly suggested in the development of narcolepsy [83]. A recent meta-analysis shows that serum levels of IL-6 and TNF-α were higher in all narcoleptic patients than in control patients [83,85]. As discussed earlier, intracerebral administration of TNF-α induces NREMS in rats. Higher cytokine levels may account for longer sleep hours in narcoleptic patients; however, the neuronal mechanisms causing these changes are unknown. Increased inflammatory response during infection with influenza virus has been linked to a variety of sleep dysfunctions. Daytime sleepiness may be a typical symptom of many types of illness. It may be induced by or associated with production of pro-inflammatory cytokines such as IL-1 and TNF-α [86]. The pathophysiology of the bilateral thalamic necrosis observed in Japanese infants with influenza infection may also be influenced by an excessive cytokine release, or “cytokine storm” [87]. According to a recent study, the H1N1 influenza virus affects sleep–wake cycles in mice and causes narcolepsy-like abrupt sleep episodes. The brain of the infected rats in this experiment had noticeably higher quantities of transcripts for TNF-α, IL-1, and IFN- α, which are important for slow wave sleep recovery following sleep deprivation.

Sleep loss can attenuate the immune response and a compromised immune system can lead to fragmented sleep [85]. A crucial crosstalk exists between NA and inflammatory cytokines during sleep disturbances. During sleep loss, the sympathetic nervous system releases NA into primary and secondary lymphoid organs. The NA stimulates adrenergic receptors in leukocytes and activates nuclear factor-κB (NF-κB) in the basal forebrain, lateral hypothalamus, and cerebral cortex [85]. The activation of NF-κB stimulates the secretion of IL-6 and TNF-α. As the circulating levels of cytokines increase, it can cause neuro-inflammation and increases the risk of damaging neural tissue. Thus, as optimum REMS is expected to maintain the brain level of NA, we propose that by maintaining optimum levels of NA, REMS also maintains the optimum immune response. The crucial interplay of microglia (immune cells of the brain), sleep (including REMS), and NA will be elaborated in the next sections.

## 4. Microglia Activation and Noradrenergic System

The nervous system requires immune cells to fight foreign body invasion including the pathogenic infection. This function is accomplished by microglia, which also play many other roles in the central nervous system including elimination of apoptotic cells, synaptic pruning, supporting neuronal survival, clearing debris, etc. [12]. Under healthy conditions, cyto-morphologically, microglia possess long thin processes and a small cell body, useful for debris clearance. However, upon exposure to inflammation, it becomes “activated” to function as immune cells of the central nervous system and develop short, thick processes and a larger cell body. They phagocytose the pathogens, release inflammatory mediators, and regulate T-cell activity. A variety of neurotransmitter receptors are expressed on the microglia which facilitate bidirectional communication between neurons and microglia. In the following subsections, we review evidence supporting the neuromodulatory role of NA in microglia activation and facilitating immune functions.

### 4.1. Role of NA in Microglial Activation

**Figure 1:**
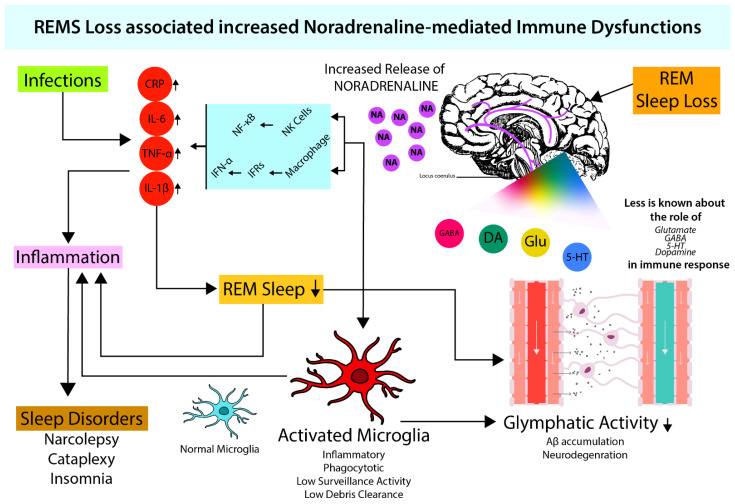
REMS maintains optimum NA levels in brain. During sleep (which also includes REMS) loss, there is an increased NA release in the brain (Mehta et al., 2017) which mediates macrophages and NK-cell activity. Increased macrophage activity leads to increased cytokine levels (IL-6, TNF-α, IL-1β) via NF-κB signaling resulting in inflammation (Sugama & Kakinuma, 2021; Sugama et al., 2019). Both increased cytokine levels and noradrenaline provide favorable conditions for microglial activation (Liu et al., 2019). Activated microglia leads to attenuated glymphatic activity (Benveniste et al., 2019; Leng et al., 2021), which elevates neuroinflammation and causes neurodegeneration. Neuroinflammation-induced cytokines further inhibit sleep (Okun et al., 2004; Kheirandish-Gozal & Gozal, 2019).

Recent reports suggest the role of NA as a key modulator of microglial activities. Various types of adrenergic receptors (ARs) are present on microglia. However, interestingly, it has been reported that although resting microglia primarily expresses β2 ARs, under proinflammatory conditions they can express α2A ARs as well [88]. Activation of microglial β2 ARs by NA downregulates the expression of proinflammatory genes [89], whereas α2A activation upregulates proinflammatory cytokines. Therefore, depending on subtypes of ARs, NA can activate or inhibit the microglia [90]. Microglial activation is inhibited by pretreatment with β-AR blockers such as propranolol. Propranolol also increases microglial process surveillance activity [91]. Moreover, NA inhibits nitric oxide production from microglia and thus, attenuates free radical production. Thus, NA may modulate sensitivity of microglia to respond to tissue damage, which holds therapeutic potential against neurodegeneration (Figure 1).

Microglia under resting conditions may have constructive roles in surveillance, such as debris clearance, pruning, remodeling, and functioning of synapse. However, activated microglia may disturb the homeostasis and may appear to be self-destructive. Using pharmacological and chemogenetic approaches, it has been shown that NA signaling in awake mice suppresses microglial surveillance activity [92]. Thus, upon sleep loss (including REMS loss), increased levels of NA can cause neuroinflammation by glial activation, leading to neurotoxicity, loss of tissue integrity, and aggravated tissue damage leading to impairment of brain functions [93]. The severity of loss of function depends on quantity and quality of loss, chronicity of the condition(s), and effects of recovery.

The role of microglia in neurodegenerative diseases viz. Alzheimer’s disease (AD) and Parkinson’s disease is of late gaining global attention. Microglia are activated by amyloid beta (Aβ) accumulated in AD, and that produces IL-1 and TNF-α. Normally, this mechanism helps clear the Aβ and τ-protein aggregates by microglial phagocytosis and cytokine activity which maintains homeostasis. However, if the formation of plaques and tangles increases, significantly overwhelming the microglial response, AD sets in. Furthermore, if there is overproduction of cytokines, it leads to neurotoxicity and (neuronal and glial) cell death [94]. Thus, NA plays a key role on one hand by facilitating microglial activation and clearance of Aβ peptide by phagocytosis, and on the other hand, it prevents Aβ-induced increase in proinflammatory cytokine release [95]. As the degeneration causes loss of NA-ergic neurons, the protective care of NA is withdrawn, resulting in the loss of neuron–neuron and neuron–glia communication, microgliosis, and defective Aβ clearance leading to neurodegenerative diseases, e.g., AD [96]. This view may be supported by the fact that a low dose of NA favors neuronal survival and growth, while its high dose facilitates neuronal degeneration [23].

**Figure 2:**
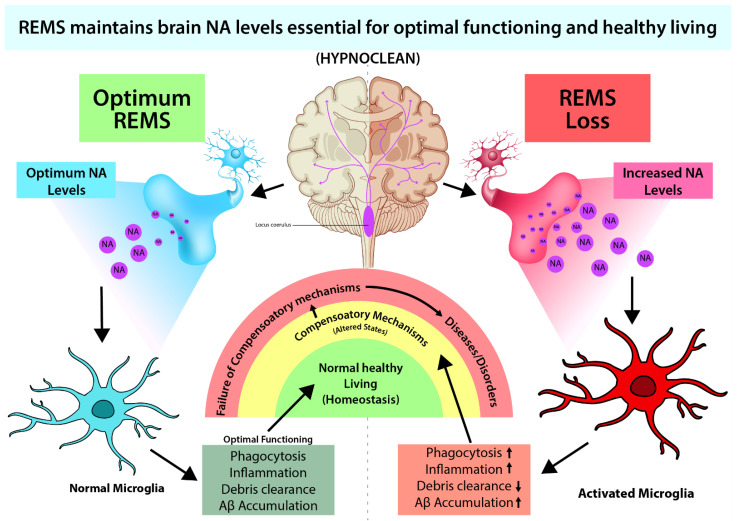
REMS loss-induced elevated brain NA causes many associated symptoms, such as immune dysfunctions, reduced waste clearance, etc., which can be ameliorated by preventing NA action (Sugama & Kakinuma, 2021; Sugama et al., 2019; Liu et al., 2019). We propose that maintaining optimum brain NA level is necessary for healthy living. We coined and introduced the term Hypnoclean to explain the overall functioning.

As NA levels tend to increase, REMS appears to bring the level of NA to protective levels and homeostasis is maintained [20]. In case of loss of sleep (including REMS loss), an initial small increase in NA activates microglia to exert protection. However, extended loss of REMS causes significant increase in NA, which is likely to affect many other systems causing damage to the brain. We propose that such significant increase in NA level in the brain might be responsible for behavioral changes particularly under chronic sleep disturbed conditions (Figure 2). Thus, dose dependent effect of NA on neuronal survivability supports our contention; the molecular action of NA on microglia in evolution needs further study. Based on such study, it has been proposed that REMS has evolved to maintain the brain level of NA [21].

### 4.2. Possible Effect of NA on the Glymphatic System through Microglia

The glymphatic system is a recently identified glia-dependent waste clearance pathway in the brain and constitutes the brain’s “front end” waste drainage system [97]. It is a transportation system that plays a significant role in the clearance of debris produced in the brain, including Aβ. Impaired glymphatic clearance has been linked to neurodegenerative diseases (Figure 1). The process of debris clearance occurs when the rapidly entering cerebrospinal fluid in the peri-arterial space exchanges with the interstitial fluid in the surrounding parenchyma. This interstitial fluid flows towards the peri-venous space, and the final exchange of debris-filled fluid occurs before drainage into the lymphatic vessels [90]. The glymphatic system has been closely linked to the sleep–wake cycle. As waste generation is a continuous process, its disposal is executed by a normal physiological process. Normal sleep is one such process that helps maintain the brain waste drainage across the lifespan of an individual [98]. As such, lifestyle and factors affecting sleep cycle such as diet, alcohol intake, exercise, meditation, temperature, light, sleep posture, intermittent fasting, and chronic stress modulate glymphatic clearance. Its functioning has been found to differ not just in sleep and wakefulness, but also during specific stages of sleep [97]. During sleep, the interstitial fluid volume fraction increases to 23% as compared to 14% during wakefulness [99]. This was closely related to the faster glymphatic transport and waste clearance during sleep in rodents [100]. It has also been observed that glymphatic activity varies with body positions. A supine or lateral decubitus position has positive effects on debris clearance by higher glymphatic activity, whereas a prone position seems to be negatively associated with debris clearance [98].

Recent studies suggest that during sleep, the glymphatic system operates through microglial activation. Aquaporin-4 (AQP-4) is a major water channel in the central nervous system and AQP-4 is essential in exchanging cerebrospinal fluid and interstitial fluids in the perivascular space. AQP-4 is significantly expressed in astrocytes and microglia; hence, their actions may be instrumental for adequate functioning of the glymphatic system during sleep. During dementia-associated sleep loss, an age-related decline in AQP-4 polarization has been shown. Mice with a dysfunctional AQP-4 channel were not able to clear Aβ efficiently [90,97]. The basis for sleep-induced enhancement of glymphatic transport appears to be closely twined with NA-ergic [101] neuronal activity. NA release during wakefulness suppresses glymphatic clearance by decreasing the amount of interstitial space. Blocking NA release expands the interstitial volume, enhances glymphatic clearance that boosts removal of metabolic waste products from the brain, and protects the brain. Another study has shown that mice treated with a combination of dexmedetomidine (NA antagonist) and isoflurane is more effective in increasing glymphatic activity than treating them with only isoflurane. Under normal condition, as NA level is lowest in the brain during REMS, it is likely that glymphatic system would be maximally effective during this stage. Such correlations underline the importance of REMS in maintaining normal brain functioning, primarily by clearing debris (waste). Because of this, we introduce a term for function of sleep as “Hypnoclean”, i.e., to clean the brain fluid during sleep.

## 5. Conclusions and Future Direction

Sleep, particularly REMS, has evolved as a fundamental, protective mechanism crucial for maintenance of normal brain and immune functions, at least in species higher in evolution, including humans. During sleep, the fluid in the brain is filtered to remove the waste. We coined and introduced this function of sleep as “Hypnoclean”. REMS is likely to mediate its action by maintaining the level of NA in the brain. Low level of NA exerts a beneficial effect on the antioxidant compromised organ, the brain, while high dose is damaging. The NA acts both on neurons and glia, and microglia as well, and mediates its action in a dynamic manner. Therefore, maintaining sleep hygiene and sleep discipline is important to enjoy normal healthy living. As a corollary, we propose that in diseased condition, caregivers need to pay additional attention to record the sleep profile of the patient and attempt to bring them to normal level in addition to prescribing disease-specific medicines.

## Data Availability

Not applicable.

## Abbreviations

ARs	adrenergic receptors
AD	Alzheimer’s disease
AQP-4	aquaporin-4
CRP	C-reactive protein
GABA	gamma-amino butyric acid
ILs	Interleukins
IL-6	interleukin-6
IL-1β	interleukin-1 β
NA	Noradrenaline
NF-κB	nuclear factor-κB
NREMS	non-rapid eye movement sleep
REMS	rapid eye movement sleep
TNF-α	tumor necrosis factor-α
